# Ligand complex structures in protein crystallography

**DOI:** 10.1107/S2059798317001644

**Published:** 2017-02-01

**Authors:** Judit É. Debreczeni, Paul Emsley

**Affiliations:** a Structure & Biophysics, Discovery Sciences, Innovative Medicines and Early Development Biotech Unit, AstraZeneca, 310 Cambridge Science Park, Cambridge, Cambridgeshire CB4 0WG, England; b Structural Studies, MRC Laboratory of Molecular Biology, Francis Crick Avenue, Cambridge CB2 0QH, England

**Keywords:** CCP4 Study Weekend 2016, protein–ligand complexes, protein crystallography

## Abstract

An introduction to the Proceedings of the 2016 CCP4 Study Weekend on protein-ligand complex structures.

Since the very early days of protein crystallography, small-molecule ligands have routinely accompanied protein molecules; indeed the first structures seen through the eyes of macromolecular crystallography contained heme moieties associated with hemoglobin and myoglobin.

The Protein Data Bank accepted the first structure depositions in the early 1970s and the number of unique ligand molecules rose to about 20 000 in the last half a century. Ligand molecules range from simple ions to large and complex cofactors, elaborate drug-like compounds and intricate post-translational modifications. In spite of their crucial role in biochemical processes, ligands have been the stepchildren of protein crystallography until recently. Unfortunately, this also shows in the quality of many of the deposited ligand structures. This discrepancy can be attributed to a somewhat slower pace of development in ligand building and validation tools within protein crystallography software packages compared with their purely macromolecular counterparts.

Over the last few years this gap has closed considerably. Improvements have been made not only as a result of the efforts made by crystallographic software developers but also because of collaborations involving macromolecular and small-molecule crystallographers, computational chemists and the wwPDB. At the same time, the generation of ligand-complex structures in a high-throughput fashion has gained ground in academic settings as well as at central facilities such as synchrotrons. This in turn presented a higher demand for more robust and automated pipelines that are able to handle ligands and also provided a fertile ground for further improvements and collaborations. The most crucial development of recent years within the *CCP*4 software suite was the novel approach to restraint dictionary generation and the release of the program *AceDRG*. To promote progress further, outside of *CCP*4, experts of protein-ligand structure determination and exploitation convened to devise best practices for validation, publication and representation of these structures.

Following these rapid changes, crystallography of protein–ligand complex structures was chosen as the topic for the CCP4 Study Weekend held in Nottingham 8–10 January 2016. In spite of the deviation from more traditional topics for these meetings, the 2016 Study Weekend attracted a large inter­national audience with a higher than average attendance, especially amongst students.

Beyond providing a forum for software developers in the ligands field to showcase their latest contributions, the meeting also gave an overview to novice students of the ligand-complex structure determination process from sample preparation through to model building and structure exploitation. Advances both in hardware and software pipelines were presented. Strong common themes included the rich legacy available in the Protein Data Bank, interdisciplinary collaborations and the use of complementary techniques. Stephen Burley and Bernhard Rupp gave introductions from two distinct points of view: one focusing on the validation of already existing data, the other outlining future plans for ligand structures in the Protein Data Bank. Ilka Müller and Patrick Collins presented different ways of generating samples for ligand studies. Roberto Steiner, Julie Tucker and Garib Murshudov took the audience through the nitty-gritty details of obtaining high-standard restraints dictionaries for refinement. Rob Nicholls, Joana Pereira, Nicholas Pearce and Paul Adams presented new developments to aid ligand placement into electron-density blobs. Paul Emsley wowed the audience with organic small-molecule validation tools, whereas validation of carbohydrate and metal ligands were discussed by Jon Agirre and Heping Zheng. Ben Bax, Gregory Warren and Oliver Smart highlighted the importance of getting the chemistry and hydrogen placement right, and Peter Moody presented experimental ways to do so. Jason Cole and Colin Groom from the Cambridge Crystallographic Data Centre discussed synergies between small-molecule and protein crystallography. In the final session Charlotte Deane and Martin Noble demonstrated ways of generating and exploiting large numbers of protein–ligand complex structures.

These proceedings contain topical review and research articles based on presentations at the CCP4 Study Weekend 2016. We hope that the concepts and tools described here will contribute to further improvements in both the tools that generate protein–ligand complex structures and the resulting deposited structures themselves.

The scientific organisers are grateful to CCP4 staff and Working Groups for their practical help and advice in the shaping of the 2016 Study Weekend, and the speakers for their outstanding contributions.

